# Intracellular Density of *Wolbachia* Is Mediated by Host Autophagy and the Bacterial Cytoplasmic Incompatibility Gene *cifB* in a Cell Type-Dependent Manner in Drosophila melanogaster

**DOI:** 10.1128/mBio.02205-20

**Published:** 2021-01-12

**Authors:** Mark Deehan, Weiwei Lin, Benjamin Blum, Andrew Emili, Horacio Frydman

**Affiliations:** a Department of Biology, Boston University, Boston, Massachusetts, USA; b Center for Network Systems Biology, Boston University, Boston, Massachusetts, USA; c National Emerging Infectious Diseases Laboratories, Boston University, Boston, Massachusetts, USA; Instituto Gulbenkian De Ciencia; University of Hawaii at Manoa

**Keywords:** *Wolbachia*, autophagy, effector functions, host-pathogen interactions, innate immunity, symbiosis

## Abstract

Autophagy is an intracellular degradation pathway involved in innate immunity. Pathogenic bacteria have evolved several mechanisms to escape degradation or exploit autophagy to acquire host nutrients. In the case of endosymbionts, which often have commensal or mutualistic interactions with the host, autophagy is not well characterized. We utilized tissue-specific autophagy mutants to determine if *Wolbachia,* a vertically transmitted obligate endosymbiont of Drosophila melanogaster, is regulated by autophagy in somatic and germ line cell types. Our analysis revealed core autophagy proteins Atg1 and Atg8 and a selective autophagy-specific protein Ref(2)p negatively regulate *Wolbachia* in the hub, a male gonad somatic cell type. Furthermore, we determined that the *Wolbachia* effector protein, CifB, modulates autophagy-*Wolbachia* interactions, identifying a new host-related pathway which these bacterial proteins interact with. In the female germ line, the cell type necessary for inheritance of *Wolbachia* through vertical transmission, we discovered that bulk autophagy mediated by Atg1 and Atg8 positively regulates *Wolbachia* density, whereas Ref(2)p had no effect. Global metabolomics of fly ovaries deficient in germ line autophagy revealed reduced lipid and carbon metabolism, implicating metabolites from these pathways as positive regulators of *Wolbachia*. Our work provides further understanding of how autophagy affects bacteria in a cell type-dependent manner.

## Abstract (precis)

Autophagy is a eukaryotic intracellular degradation pathway which can act as an innate immune response to eliminate pathogens. Conversely, pathogens can evolve proteins which modulate the autophagy pathway to subvert degradation and establish an infection. *Wolbachia*, a vertically transmitted obligate endosymbiont which infects up to 40% of insect species, is negatively regulated by autophagy in whole animals, but the specific molecular mechanism and tissue which govern this interaction remain unknown.

## INTRODUCTION

*Wolbachia* is a widespread obligate endosymbiont, estimated to infect up to 40% of terrestrial arthropods as well as filarial nematodes (1, 2). Recently, *Wolbachia* has been employed as a biocontrol agent to reduce the spread of several human diseases, including Zika virus infection, dengue virus infection, and malaria (3–5). This is accomplished through artificial transfection of *Wolbachia*-uninfected mosquitoes with a *Wolbachia* strain, *w*Mel, originating from the fruit fly Drosophila melanogaster (6, 7). While the exact mechanisms eliciting pathogen blocking by *Wolbachia* remain unknown, *Wolbachia* density has been correlated with the effectiveness of this process (8–10). Several studies have begun to elucidate host-related pathways which modulate intracellular *Wolbachia* density, postulating an indirect means to boost pathogen blocking (11–15). Utilizing genetic analysis in Brugia malayi and the autophagy-inducing drug rapamycin in Drosophila melanogaster larvae, autophagy was identified as a negative regulator of *Wolbachia* (16). Interestingly, when *Wolbachia* was investigated in the female germ line after adult flies were fed rapamycin, *Wolbachia* levels were increased (17). This indicates a possible cell type-dependent effect that autophagy may have in regulating *Wolbachia* density. It should be noted that rapamycin inhibits target of rapamycin (TOR) signaling, which regulates several downstream signaling pathways, including those that mediate cell growth, protein translation, and autophagy (18). Genetic manipulations to confirm that autophagy is responsible for modulating *Wolbachia* densities in this cell type-dependent manner remain incomplete.

Macroautophagy, here referred to as autophagy, is a well-conserved eukaryotic degradation and recycling pathway used to protect the cell from different stresses, including nutritional starvation, mitochondrial damage, and intracellular pathogens (19, 20). Autophagy is characterized by the formation of an autophagosome, a double-membrane vesicle, which sequesters and shuttles cargo to the lysosome for degradation (21). There are two main forms of autophagy, selective and bulk, where the former is active under physiological normal conditions and maintains cellular homeostasis and the latter responds to nutrient starvation to recycle cytoplasmic constituents for the cell to use (21–23). Selective and bulk autophagy utilize a similar set of core autophagy genes to form an autophagosome (24). Atg1, a serine/threonine kinase, is the most upstream protein and is known to induce the formation of an autophagosome (25). Atg8 is a ubiquitin-like protein found conjugated to phosphatidylethanolamine (PE) and incorporated into autophagosome membranes and is essential for autophagosome formation (26). *Drosophila* have two Atg8 genes: the well-characterized and ubiquitously expressed Atg8a and its paralog Atg8b, which is restricted in expression to larval developmental stages and adult male testis (27). During selective autophagy, Atg8 is capable of binding to the adaptor protein Ref(2)p (p62 in mammals), which can target specific cargo to autophagosomes for degradation (28, 29). Selective autophagy has been well characterized as an innate immune response to pathogenic bacteria and viruses, but its role in regulating endosymbionts is less understood.

Bacteria have evolved several ways to evade host autophagy and ensure survival. One method, deubiquitination, allows for bacteria to escape recognition by host selective autophagy adapter proteins and thus survive in the cell. *Salmonella* and *Chlamydia* secrete the deubiquitinating (DUB) enzymes Ssel and ChlaOTU and ChlaDUB, respectively, which target and remove ubiquitin (30–32). Interestingly, *Wolbachia* has been shown to harbor a deubiquitinase named cytoplasmic incompatibility factor B (CifB), which is sufficient to drive the reproductive phenotype of cytoplasmic incompatibility (33–35).

Cytoplasmic incompatibility (CI) is a reproductive phenomenon observed when a *Wolbachia*-infected male mates with an uninfected female, rendering the eggs sterile. It has been discovered that when *Drosophila* uninfected with *Wolbachia* express two *Wolbachia* proteins, CifA and CifB, in the male germ line, CI can be recapitulated (33, 36). Biochemical studies utilizing the CifB homologue, CidB, from *Wolbachia* infecting the mosquito Culex pipiens (*w*Pip) showed that a functional DUB domain was necessary to drive CI when expressed in Drosophila melanogaster (34). Interestingly, CifA can act as a rescue factor, whereby it inhibits CI when expressed in the female germ line. This reveals a complex and incomplete mechanism, where CifA aids CifB in driving CI when expressed in the male germ line but CifA rescues this effect when expressed in the female germ line.

Here, we utilized genetic manipulation of core autophagy genes (*atg1* and *atg8a*) and the selective autophagy gene *ref(2)p* to determine if *Wolbachia* density is affected by autophagy in an opposite cell type-dependent manner as previously suggested (16, 17). We first analyzed autophagy’s effect on *Wolbachia* density in the hub, a nondividing somatic cell type in which *Wolbachia* density has been extensively characterized (37). The hub is established during embryonic development, anchors both the somatic and germ line stem cells, and regulates stem cell division and differentiation (38, 39). We determined that *Wolbachia* is negatively regulated in the hub by Ref(2)p-dependent selective autophagy in a strain-dependent manner. Additionally, overexpression of the two *Wolbachia* effector proteins, CifA and CifB, implicate CifB, a bacterial DUB, in positively regulating bacterial density in the hub. Epistasis analysis between CifB and autophagy protein Atg1 provides evidence they are acting in the same pathway.

Conversely, in the female germ line, *Wolbachia* density is positively affected by autophagy in a strain-independent manner, and Ref(2)p-mediated selective autophagy has no effect in regulating *Wolbachia* density. This suggests that *Wolbachia* utilizes a bulk autophagy program to increase its bacterial load. To begin to identify what metabolites autophagy modulates to aid in *Wolbachia* growth, we utilized a global metabolomics analysis. This analysis allows us to identify changes in host metabolic pathways at the level of metabolites, whereas RNA sequencing or proteomics may not directly reflect metabolic shifts caused by a knockdown of autophagy. Global metabolomics of autophagy mutants in the female germ line reveal a downregulation of glycolysis and glycerolipid metabolism, implicating metabolites from this pathway as positive regulators of *Wolbachia*. Together, our findings demonstrate a mechanism by which a *Wolbachia* effector protein and host autophagy proteins act in regulating bacterial density in a cell type- and strain-dependent manner.

## RESULTS

### Autophagy negatively regulates *Wolbachia w*Mel but not *w*MelCS density in the hub.

**FIG 1:**
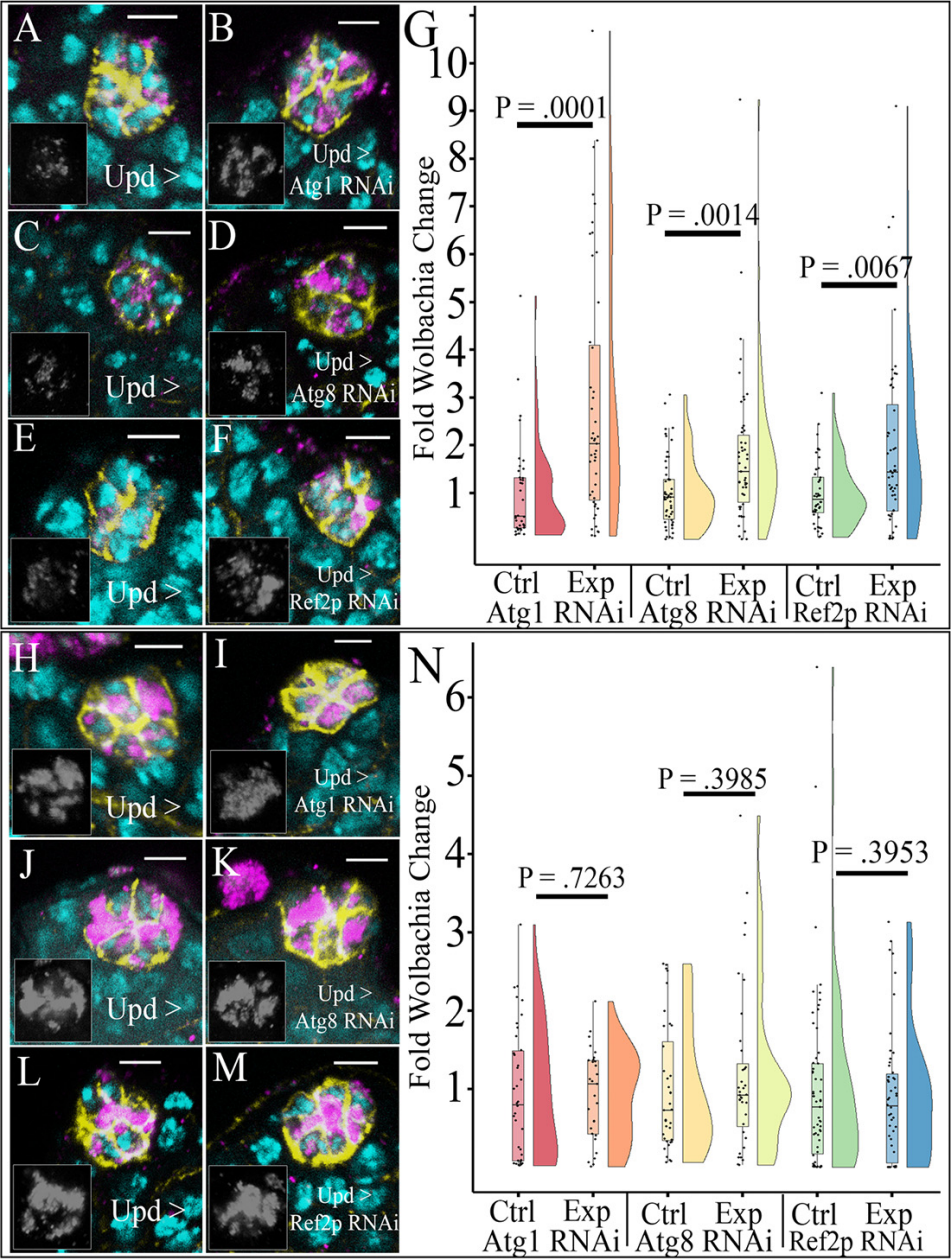
Knockdown of selective autophagy increased density of *Wolbachia* strain *w*Mel but not *w*MelCS in the hub. Representative confocal z-stacks of hubs expressing RNAi against autophagy genes. Unpaired (Upd) was used to express small interfering RNAs (siRNAs) specifically in the hub and not male germ line and soma in the testis. DNA is colored cyan, D cadherin (labeling the hub) is yellow, and the HSP60 antibody detecting *Wolbachia* is in magenta. The insets display grayscale images of only the *Wolbachia* channel from the respective image. (A) Sibling control hub of *w*Mel-infected male testis displaying *Wolbachia* at a low density. (B) Knockdown of Atg1 increased *w*Mel density in the hub. (C) Control Atg8 RNAi *w*Mel-infected male hub displaying low *Wolbachia* density. (D) Knockdown of Atg8 in the hub increased *w*Mel density. (E) Control Ref(2)p RNAi hub of *w*Mel-infected male testis where *Wolbachia* is at a low density. (F) Knockdown of Ref(2)p with RNAi increases *Wolbachia w*Mel density in the hub. (G) Vertical raincloud plots display each quantified value overlaid on a box and whisker plot showing the median value, upper and lower quartiles (box), and upper and lower extremes (whiskers, 1.5× interquartile range). A split violin plot accompanies each box and whisker plot which displays the probability density function of the data set. Quantification of relative *Wolbachia* density reveals a significant increase in *Wolbachia* density upon knockdown of either Atg1 (*N*^cont^ = 37, *N*^exp^ = 47), Atg8 (*N*^cont^ = 54, *N*^exp^ = 50), or Ref(2)p (*N*^cont^ = 44, *N*^exp^ = 52) in the hub. (H) Control Atg1 RNAi hub of *w*MelCS (CS)-infected male testis where *Wolbachia* is at a moderate/high density. (I) Knockdown of Atg1 has no significant effect on moderating the density of CS. (J) Control Atg8a RNAi hubs of CS-infected male testis display a moderate/high density. (K) Atg8a knockdown displays a moderate/high density of CS similar to that for the control. (L) Control Ref(2)p hub of *w*MelCS-infected hubs displays moderate density. (M) Knockdown of Ref(2)p does not result in any change in *w*MelCS density, displaying moderately infected hub densities. (N) Quantification of relative CS density in the hub shows no difference in density upon knockdown of Atg1 (*N*^cont^ = 39, *N*^exp^ = 31), Atg8 (*N*^cont^ = 36, *N*^exp^ = 36), or Ref(2)p (*N*^cont^ = 59, *N*^exp^ = 50). Scale bars, 5 µM. Mann-Whitney U tests were performed for statistical analysis.

To determine the effect autophagy has in regulating *Wolbachia* density in somatic tissues, we knocked down core autophagy proteins Atg1 and Atg8a in the *Drosophila* hub, a cell type previously shown to have high levels of *Wolbachia* tropism (37). Knockdown of Atg1 resulted in a marked increase in *Wolbachia w*Mel accumulation in the hub (Fig. 1A and B). Quantification of relative *Wolbachia* density showed a 2.87-fold increase in average *Wolbachia* density upon Atg1 knockdown (Fig. 1G). To confirm the autophagy pathway was involved in regulating hub cell *Wolbachia* density, we knocked down Atg8a. We specifically targeted *atg8a* because it is much more extensively characterized and ubiquitously expressed in the male and female tissues we were investigating than *atg8b*. Upon knockdown of Atg8a, we saw an increase in the density of *w*Mel (Fig. 1C and D). Quantification of relative *Wolbachia* density showed a 1.77-fold increase upon Atg8a knockdown (Fig. 1G).

*w*MelCS, a closely related strain of *Wolbachia* which resides at higher density than *w*Mel, has been shown to decrease *Drosophila* susceptibility to death compared to that with *w*Mel from the *Drosophila C virus*, while both strains induce similar levels of cytoplasmic incompatibility (40, 41). These observations highlight strong phenotypic variability even in closely related bacterial strains. We hypothesized that *w*MelCS would also be affected by autophagy due to the minimal genomic differences from *w*Mel (41). Upon knockdown of Atg1, we saw no noticeable difference in *w*MelCS density in the hub (Fig. 1H and I). Quantification showed no discernible difference in densities infecting the hubs, where there was a near even 1.02-fold decrease in average *w*MelCS density (Fig. 1N). Atg8a knockdown also resulted in no change in *w*MelCS density within the hub (Fig. 1J and K). Quantification of hubs expressing Atg8a RNA interference (RNAi) showed a nonsignificant 1.18-fold increase in average density of *w*MelCS (Fig. 1N). It should be noted that there was no difference in *Wolbachia* tropism (the preferential accumulation of *Wolbachia* in a specific cell type or tissue) upon modulation of either Atg1 or Atg8a (see Fig. S2A and B in the supplemental material). Lastly, knockdown of Atg1 and Atg8 resulted in increased levels of Ref(2)p protein in the hub compared to that for control staining, indicating an efficient knockdown of autophagy (Fig. S1A to D).

10.1128/mBio.02205-20.1 FIG S1 Ref(2)P staining confirms RNAi knockdown of autophagy genes. Ref(2)p is increased in tissues with autophagy knocked down. Image of hubs labeled with genotypes (control on the left, experimental on right) antibody stained for D-cadherin (yellow) and Ref(2)p (magenta). Prime-labeled panels are greyscale of Ref(2)p staining. (A) Control hub displays few small Ref(2)p puncta within the hub. (B) Atg1 RNAi expressed in the hub causes several smaller Ref(2)p puncta. (C) Control flies display several small Ref(2)p puncta within the hub. (D) Expression of Atg8a RNAi in the hub causes an increase in Ref(2)p puncta and size, indicating a block in autophagy. (E, E′) Control flies show a few small Ref(2)p puncta in the hub. (E′) Grayscale of the Ref(2)p channel is shown. (F) Ref(2)p RNAi knocks down expression of Ref(2)p in the hub. (F′) Grayscale image of Ref(2)p channel shows little to no expression of Ref(2)p in the hub. Scale bars, 5 µM. Download FIG S1, PDF file, 1.3 MB. Copyright © 2021 Deehan et al. 2021 Deehan et al. This content is distributed under the terms of the Creative Commons Attribution 4.0 International license.

10.1128/mBio.02205-20.2 FIG S2 Autophagy does not affect *w*Mel and CS hub tropism. (A) Knockdown of either Atg1 or Atg8a does not affect the proportions of hubs displaying tropism for strain *w*Mel. (B) Knockdown of Atg1 or Atg8a does not affect the proportions of hubs displaying tropism for the strain CS. (C) Ref(2)p knockdown does not affect hub tropism proportions in either *w*Mel or CS. *P* values represent proportions test between control and knockdown. Error bars represent 95% confidence intervals. Download FIG S2, PDF file, 2.0 MB. Copyright © 2021 Deehan et al. 2021 Deehan et al. This content is distributed under the terms of the Creative Commons Attribution 4.0 International license.

### The selective autophagy adapter protein Ref(2)p negatively regulates *Wolbachia w*Mel but not *w*MelCS in the hub.

Selective autophagy has been implicated in regulating several mammalian intracellular bacterial infections (42, 43). We wanted to test if Ref(2)p-mediated selective autophagy was involved in regulating *Wolbachia* densities in the hub. Upon knockdown of Ref(2)p in the hub, we saw an increase in *w*Mel density (Fig. 1E and F). Quantification of relative *Wolbachia* density in the hub showed an average 1.94-fold increase in *w*Mel density upon knockdown of Ref(2)p (Fig. 1G).

While our previous data revealed no significant effect of autophagy in regulating *w*MelCS density, we wanted to confirm that selective autophagy does not affect *w*MelCS as well. Indeed, Ref(2)p knockdown did not affect *w*MelCS density in the hub. Representative images displayed no change in hub density in the control or Ref(2)p knockdown (Fig. 1J and K). Quantification of hubs showed an average 1.13-fold decrease in *w*MelCS density (Fig. 1N). To verify the knockdown of Ref(2)p in the hub, we performed antibody staining and saw reduced Ref(2)p puncta formation compared to that in control hubs (Fig. S1E and F). It should be noted that there was no difference in *Wolbachia* tropism upon modulation of Ref(2)p (Fig. S2C).

### *Wolbachia* effector protein CifB modulates bacterial density of *w*Mel in the hub.

To determine if *w*Mel harbored factors could play a role in regulating *w*Mel density, and because *Wolbachia* is currently not able to be transformed, we used the Gal4-upstream activation sequence (UAS) system to overexpress (OE) either CifA, CifB, or CifA and CifB together in hub cells. We hypothesized that the similar deubiquitinating activities of CifB and CidB would aid *Wolbachia* in escape from ubiquitination, detection, and thus destruction by the autophagy system (34, 35, 44).

**FIG 2:**
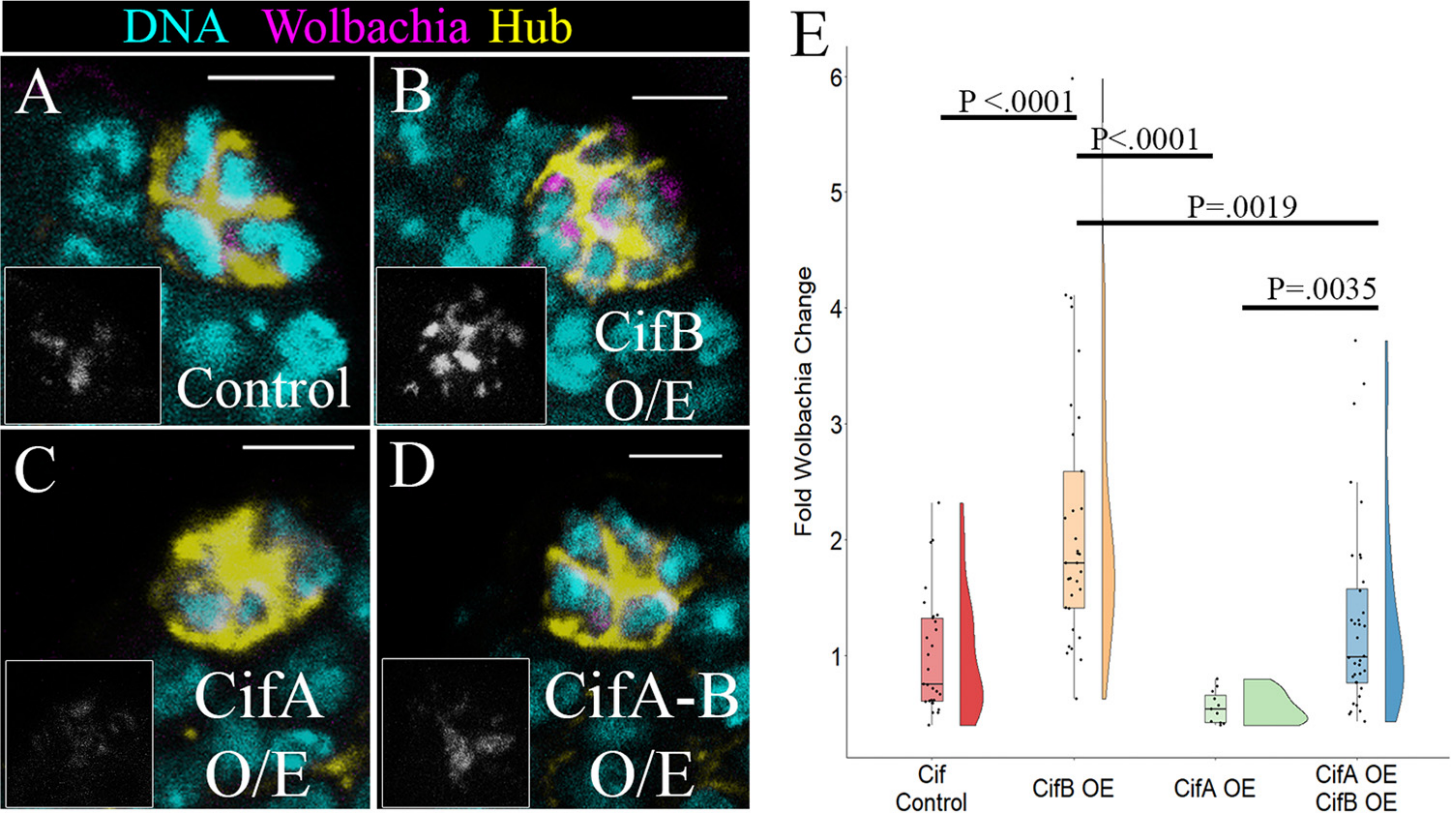
Expression of *Wolbachia* cytoplasmic incompatibility genes CifA and CifB modulate *w*Mel density in the hub. Representative confocal z-stacks of hubs with overexpressed *Wolbachia* Cif genes. Unpaired (Upd) was used to overexpress Cif constructs specifically in the hub and not male germ line and soma in the testis. DNA is cyan, D cadherin (labeling the hub) is yellow, and a fluorescently labeled DNA probe to detect *Wolbachia* is in magenta. Grayscale insets display the *Wolbachia*-only channel. (A) Sibling control hubs displayed low relative *w*Mel density. (B) Overexpression of CifB resulted in higher relative *w*Mel density. (C) Overexpression of CifA in the hub resulted in a trend for lower relative *w*Mel density. (D) Overexpression of both CifA and CifB resulted in *w*Mel hub density similar to that of the control. (E) A Kruskal-Wallis test of significance revealed a significant difference in the data set (*P* < 0.0001). Quantification of relative *w*Mel hub density showed overexpression of CifA results in a nonsignificant trend for lower *w*Mel densities (*N*^cont^ = 29, *N*^CifA^ = 11, *P* < 0.0905). Overexpression of CifB results in a significant increase in *w*Mel density compared to that in the control (*N*^cont^ = 29, *N*^CifB^ = 33, *P* < 0.00001) and to those with both CifA and CifB overexpression (*N*^CifB^ = 33, *N*^cifA-B^ = 36, *P* < 0.0019). Overexpression of both CifA and CifB resulted in no difference from the control (*N*^cont^ = 29, *N*^CifA-B^ = 36, *P* = 1.0). *P* values reported for individual comparisons were from a Kruskal-Wallis *post hoc* Dunn’s test with Bonferroni correction. Scale bars, 5 µM.

Representative images show CifB overexpression significantly increased *w*Mel density compared to that in the control (Fig. 2A and B). Quantification of *w*Mel density revealed a 2.39-fold increase in average hub *Wolbachia* density compared to that in the control (Fig. 2E). Overexpression of CifA resulted in a trend toward reduced *Wolbachia* density compared to that in control flies but did not reach significance (Fig. 2A and C). Quantification showed a decrease of approximately 1.41-fold in *Wolbachia* density in the hub (Fig. 2E). Lastly, coexpression of CifA and CifB resulted in no difference in *Wolbachia* density compared to that in the control (Fig. 2A and D). Quantification showed a 1.31-fold increase in density, but this was not significantly different from the control (Fig. 2E). Interestingly, CifB was significantly different compared to coexpression of CifB and CifA in the hub (Fig. 2B, D, and E). Overall, these results show that CifB acts to positively regulate *Wolbachia* density in the hub and suggests CifA may partially negatively regulate *Wolbachia* density.

### Autophagy-CifB epistasis reveals they function in the same pathway.

CifB contains a deubiquitinating domain, suggesting it may aid in removal of ubiquitin and help subvert autophagy. To characterize how *Wolbachia cifB* expression regulates intracellular density, we used epistasis to determine if CifB modulates density through autophagy or an independent host pathway. An additive epistasis model was tested between the *Wolbachia* gene CifB and Atg1. As previously observed, we expected an approximately 2-fold increase when only CifB was expressed and a 3-fold increase when only Atg1 RNAi was expressed. If both are expressed in the same hub and we see an approximately 5-fold increase, then both constructs are working in independent pathways to modulate *Wolbachia* density. If the hub *Wolbachia* density phenotype is similar to that of one of the constructs, then that would reveal that these genes work in the same pathway.

**FIG 3:**
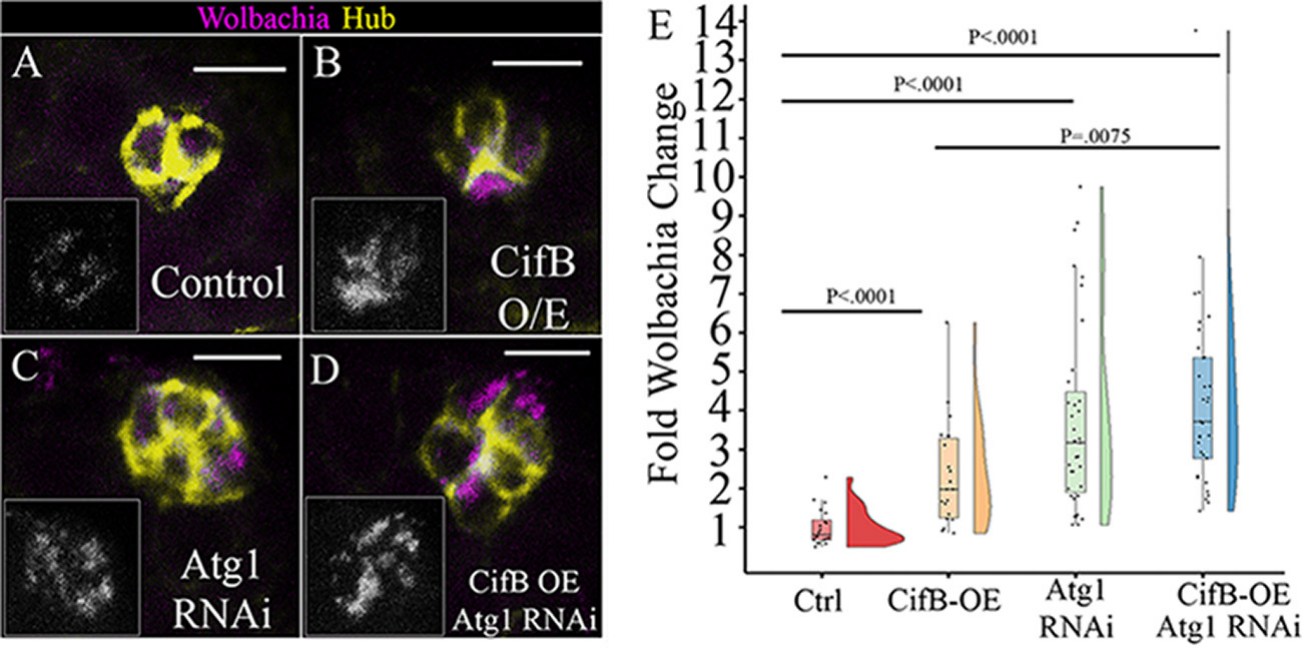
Epistasis analysis of *Wolbachia* CifB and Atg1 genes reveal *Wolbachia* effector CifB acts in the autophagy pathway. Representative confocal z-stacks of hubs. Unpaired (Upd) was used to express siRNAs or Cif constructs specifically in the hub and not male germ line and soma in the testis. DNA was not acquired for this experiment; D cadherin labeling the hub is yellow, and a fluorescently conjugated DNA probe to detect *Wolbachia* is in magenta. Grayscale insets display the *Wolbachia*-only channel. (A) Control hubs displayed low relative *w*Mel density. (B) Overexpression of CifB resulted in higher relative *w*Mel density. (C) Expression of Atg1 RNAi in the hub resulted in higher relative *w*Mel density. (D) Overexpression of both CifB and Atg1 RNAi resulted in high *w*Mel hub density, similar to that with Atg1 RNAi. (E) A Kruskal-Wallis test of significance revealed a significant difference in the data set (*P* < 0.0001). Quantification of relative *w*Mel hub density showed overexpression of CifB results in a statistically significant increase in *w*Mel density (*N*^cont^ = 20, *N*^CifB^ = 22, *P* < 0.0001). Expression of Atg1 RNAi result in a significant increase in *w*Mel density (*N*^cont^ = 29, *N*^Atg1RNAi^ = 35, *P* < 0.0001). Coexpression of CifB and Atg1 RNAi results in a significant increase in *w*Mel density compared to that in the control (*N*^Cont^ = 29, *N*^CifB-Atg1RNAi^ = 33, *P* < 0.0001) and with CifB alone (*N*^CifB-Atg1RNAi^ = 33, *N*^CifB^ = 22, *P* < 0.0075) and a density similar to that with Atg1 RNAi expression alone (*N*^Atg1RNAi^ = 35, *N*^CifB-Atg1RNAi^ = 33, *P* = 1.0). *P* values reported for individual comparisons were from a Kruskal-Wallis *post hoc* Dunn’s test with Bonferroni correction. Scale bars, 5 µM.

When we overexpressed CifB, there was a significant 2.33-fold increase in average *w*Mel hub density (Fig. 3A, B, and E). When Atg1 RNAi was expressed in hubs, there was a significant 3.89-fold increase in *Wolbachia* density (Fig. 3A, C, and E). When both constructs were expressed in the hub, we again saw an increase in *w*Mel density (Fig. 3A and D), and quantification revealed a 4.3-fold increase in *w*Mel density, which was significantly different from that in the control (Fig. 3E). Compared to that with CifB alone, coexpression of Atg1 RNAi-CifB OE was also significantly different (Fig. 3E). Compared to that when Atg1 RNAi was expressed alone, there was no statistical difference between the groups (Fig. 3E). This genetic analysis supports that CifB acts in the autophagy pathway. If they acted in different pathways, we would expect to see an additive effect of an approximately 6.22-fold increase from the control, as that would be the sum of CifB (2.33-fold) and Atg1 RNAi expression (3.89-fold) together, and this would lead to CifB-Atg1 RNAi coexpression being significantly different than both CifB and Atg1 RNAi alone.

### Autophagy positively regulates *Wolbachia* density in the female germ line.

**FIG 4:**
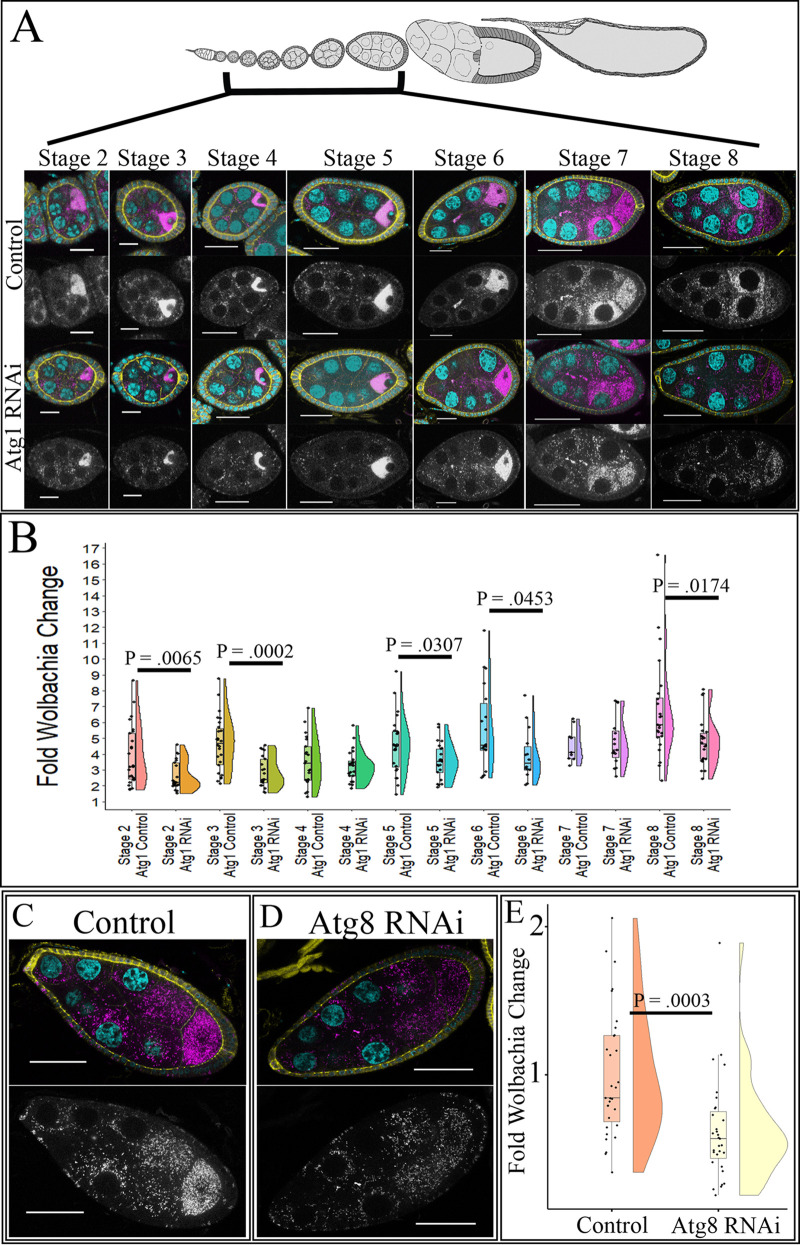
Knockdown of autophagy in the female germ line decreases *w*Mel density. Stage-specific confocal analysis reveals a decrease in relative *Wolbachia* density upon knockdown of autophagy genes during multiple stages of development. NGT;nos was used to knockdown Atg1, and MTD was used to knockdown Atg8a. DNA is colored cyan, D cadherin (labeling the follicle cells) is yellow, and a fluorescently conjugated DNA probe detecting *Wolbachia* is in magenta. Grayscale images display the *Wolbachia*-only channel. (A) Representative confocal z-stack images of control and Atg1 knockdown in the germ line of *w*Mel-infected flies. (B) Quantification of *w*Mel-infected stage-specific egg chambers upon Atg1 knockdown. Statistically significant *P* values are reported only. (C) Representative confocal z-stack images of *w*Mel-infected stage-8 control egg chambers show high levels of germ line *Wolbachia*. (D) Representative confocal z-stack image of stage-8 egg chambers with Atg8 RNAi expression displays reduced *w*Mel density in the germ line. (E) Quantification of *w*Mel-infected stage-8 egg chambers for the control and Atg8 knockdown. Scale bars, 10 µM for stages 2 and 3, 20 µM for stages 4 to 6, and 40 µM for stages 7 and 8. Student’s *t* tests were conducted to determine significance.

To investigate the effects of autophagy on *Wolbachia* density in the female germ line, we knocked down *Atg1* and *Atg8a* in only the germ line and quantified relative *Wolbachia* density in the germ line and in the surrounding follicle cells. Quantitative reverse transcriptase PCR (RT-qPCR) of whole ovaries determined that Atg1 was knocked down 77% when Atg1 RNAi was expressed under the NGT;nos-Gal4 driver (see Fig. S3A). Using confocal microscopy, the *Wolbachia* density in egg chambers between stages 2 and 8 was quantified. In *w*Mel-infected ovaries, stages 2, 3, 5, and 8 displayed 1.47-, 1.60-, 1.28-, and 1.4-fold decreases in average *Wolbachia* density, respectively (Fig. 4A and B). To confirm the role of autophagy in regulating *w*Mel density in the female germ line, we knocked down Atg8a. In this experiment, we used a stronger germ line driver, the maternal triple driver (MTD), because the knockdown efficiency utilizing NGT;nos was not >50% (Fig. S3B). Upon knockdown of Atg8a in the female germ line, we saw a 1.60-fold decrease in average relative *Wolbachia* density in stage-8 egg chambers (Fig. 4C to E). Lastly, as starvation has been shown to increase autophagy, we investigated if starvation could drive a larger difference in *w*Mel density in Atg1 RNAi ovaries. Whole-ovary qPCR analysis revealed that there was a significant decrease in the density of *w*Mel compared to that in the control, but the difference was similar to what was observed for well-fed flies (see Fig. S4).

10.1128/mBio.02205-20.3 FIG S3 Knockdown efficiency of autophagy-related mRNA in the ovaries upon RNAi expression. RT-qPCR used to determine knockdown efficiency of different RNAi constructs in whole female ovaries. Genes were normalized to RPL32 expression. (A) Two replicates of 20 ovary pools show a knockdown efficiency of 64%. (B) Three replicates of 20 ovary pools shows an Atg8a knockdown efficiency of 47% when the NGT;nos driver was used. (C) Three replicates of 20 ovary pools show a Ref(2)p knockdown efficiency of 87%. Download FIG S3, PDF file, 0.8 MB. Copyright © 2021 Deehan et al. 2021 Deehan et al. This content is distributed under the terms of the Creative Commons Attribution 4.0 International license.

10.1128/mBio.02205-20.4 FIG S4 Starvation does not modify the effect of autophagy knockdown on *Wolbachia* density in the germline. Quantitative PCR of WSP normalized to 14-3-3 of 20 whole ovaries from 10 females. (A) Whole-ovary quantitative PCR of *Wolbachia* density revealed significant decreases in *Wolbachia* density upon knockdown of Atg1 under well fed and 2-day starvation conditions. Four-day starvation resulted in a similar trend in reduced *Wolbachia* density but was not significant. *P* values represent paired Student’s *t* test results. Download FIG S4, PDF file, 1.9 MB. Copyright © 2021 Deehan et al. 2021 Deehan et al. This content is distributed under the terms of the Creative Commons Attribution 4.0 International license.

**FIG 5:**
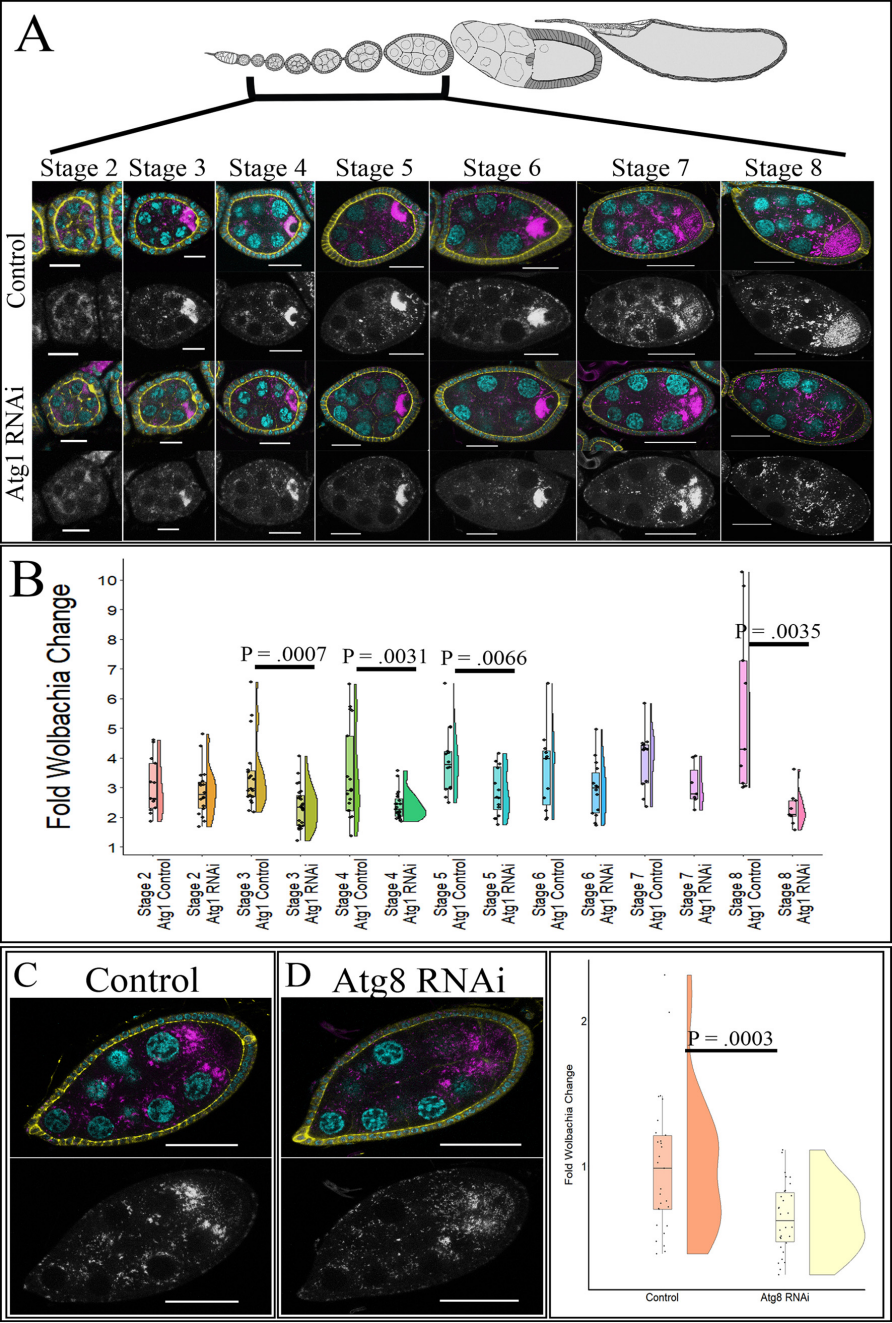
Knockdown of autophagy in the female germ line decreases *w*MelCS density. Stage-specific confocal analysis reveals a decrease in relative *Wolbachia* density upon knockdown of autophagy genes during multiple stages of development. NGT;nos was used to knockdown Atg1, and MTD was used to knockdown Atg8a. DNA is colored cyan, D cadherin (labeling the follicle cells) is yellow, and a fluorescently labeled DNA probe detecting *Wolbachia* is in magenta. Grayscale images display the *Wolbachia*-only channel. (A) Representative confocal z-stack images of control and Atg1 knockdown in the germ line of *w*MelCS-infected flies. (B) Quantification of *w*MelCS-infected stage-specific egg chambers upon Atg1 knockdown. Statistically significant *P* values are reported only. (C) Representative confocal z-stack images of *w*MelCS-infected stage-8 egg chambers for control flies display a high *Wolbachia* density. (D) Representative confocal z-stack images of *w*MelCS-infected stage-8 egg chambers with Atg8 RNAi expressed display reduced germ line *Wolbachia* density. (E) Quantification of *w*MelCS-infected stage-8 egg chambers for the control and Atg8 knockdown reveal decreased density upon germ line expression of Atg8 RNAi. Scale bars, 10 µM for stages 2 and 3, 20 µM for stages 4 to 6, and 40 µM for stages 7 and 8. Student’s *t* tests were conducted to determine significance.

Unlike the hub, autophagy knockdown in the female germ line affected *w*MelCS density as well. Upon expression of Atg1 RNAi in the germ line by the NGT;nos driver, we saw a stage-specific reduction in *w*MelCS density. Stages 3, 4, 5, and 8 displayed 1.44-, 1.44-, 1.37-, and 2.47-fold decreases in average *Wolbachia* density, respectively (Fig. 5A and B). To confirm that autophagy positively regulated *w*MelCS density in the female germ line, we used the MTD driver to express Atg8a RNAi in the germ line and quantified relative *w*MelCS levels in stage-8 egg chambers. There was a 1.55-fold reduction in average *Wolbachia* density upon Atg8a knockdown in the germ line (Fig. 5C to E).

### Ref(2)p-dependent selective autophagy does not affect *Wolbachia* density in the female germ line.

**FIG 6:**
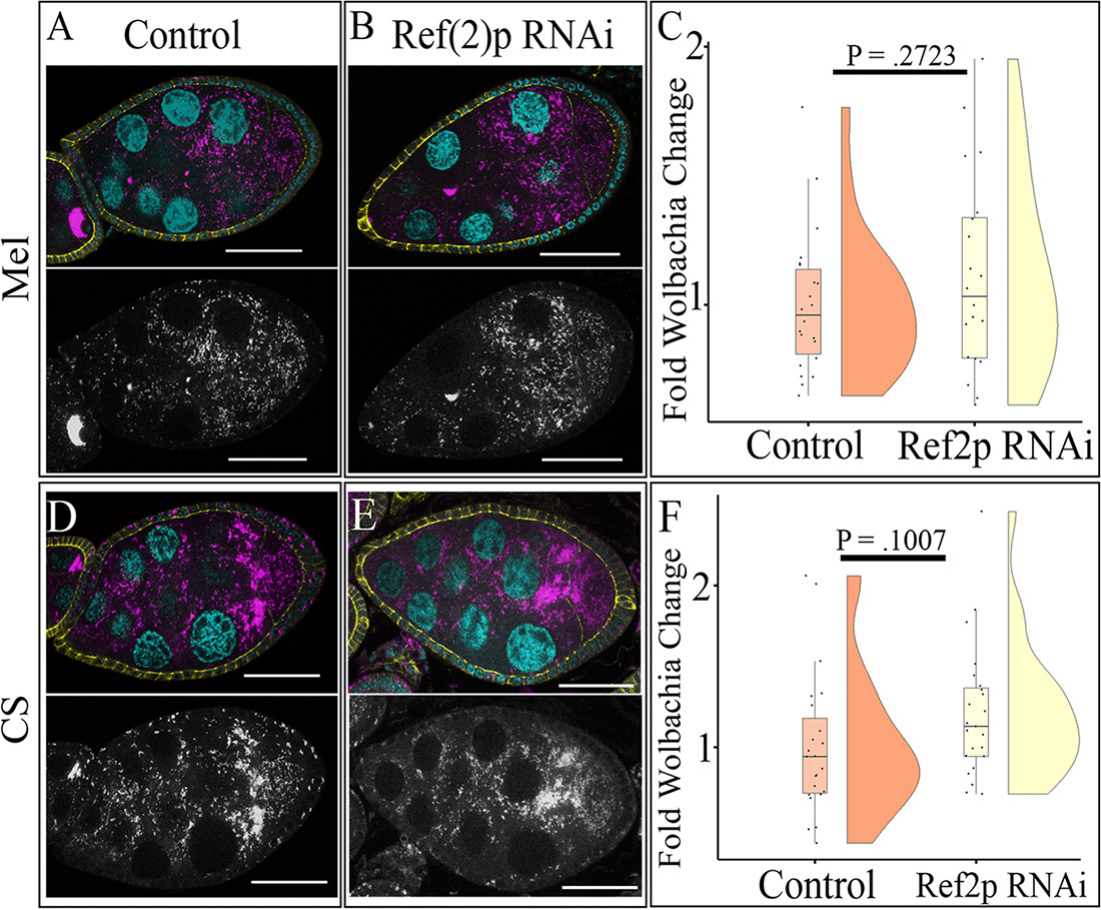
Ref(2)p does not regulate *Wolbachia* density in stage-8 egg chambers. (A) Representative confocal z-stack image of *w*Mel-infected control stage-8 egg chamber shows moderate density of *Wolbachia*. NGT;nos was used to drive knockdown of Ref(2)p. DNA is colored cyan, D cadherin (labeling the follicle cells) is yellow, and a fluorescently labeled DNA probe to detect *Wolbachia* is in magenta. Grayscale images display the *Wolbachia*-only channel. (B) Representative confocal z-stack image of *w*Mel-infected stage-8 egg chamber with Ref(2)p knocked down shows similar moderate density to that of the control. (C) Quantification of relative germ line *Wolbachia* density reveals no difference in density upon the expression of Ref(2)p RNAi. (D) Representative confocal z-stack image of *w*MelCS-infected control stage-8 egg chamber shows high germ line density. (E) Representative confocal z-stack image of *w*MelCS-infected stage-8 egg chamber with Ref(2)p knocked down displays similar germ line density to that of the control. (F) Scale bars, 40 µM. *P* values determined by Student’s *t* test.

To determine if Ref(2)p-dependent selective autophagy regulates *Wolbachia* density in the female germ line, similar to that in the hub, we knocked down Ref(2)p in the germ line and quantified relative *Wolbachia* density for both *w*Mel and *w*MelCS. Utilizing the NGT;nos driver, we were able to obtain an 86% knockdown efficiency, indicating a robust knockdown of Ref(2)p (Fig. S3C). Upon knockdown of Ref(2)p in the germ line, we saw no change in *w*Mel density in stage-8 egg chambers (Fig. 6A and B). Quantification showed a nonsignificant 1.11-fold increase in *Wolbachia* density (Fig. 6C). Whole-ovary qPCR showed a nonsignificant 1.05-fold decrease in *w*Mel density, confirming our image analysis (see Fig. S5A).

10.1128/mBio.02205-20.5 FIG S5 Quantitative PCR of Ref(2)p RNAi mutants reveals no change in *Wolbachia* density in the female germline. (A) Quantitative PCR of *w*Mel-infected ovaries reveals no change in density upon the knockdown of Ref(2)p. Five replicates of 20 ovary pools were used to determine average *Wolbachia* density. (B) Quantitative PCR of *w*MelCS-infected ovaries reveals no change in density upon the knockdown of Ref(2)p. Two replicates of 20 ovary pools were used to determine average *Wolbachia* density. Paired Student’s *t* tests were used for statistical analysis. Download FIG S5, PDF file, 0.6 MB. Copyright © 2021 Deehan et al. 2021 Deehan et al. This content is distributed under the terms of the Creative Commons Attribution 4.0 International license.

We also tested the effect of a knockdown of Ref(2)p on *w*MelCS. Image analysis revealed a nonsignificant 1.21-fold increase in *w*MelCS density in the female germ line (Fig. 6D to F). Furthermore, qPCR of whole ovaries with Ref(2)p knockdown showed a 1.07-fold decrease in *w*MelCS density (Fig. S5B). These data support a mechanism by which Ref(2)p does not influence either *w*Mel or *w*MelCS density in the germ line cell autonomously.

### *Wolbachia cif* genes do not impact *Wolbachia* density in the female germ line.

We explored whether CifA and CifB regulate *w*Mel density in the female germ line, a tissue functionally relevant for cytoplasmic incompatibility. Previous results from the hub indicated that CifB modulates *w*Mel density through autophagy. Since Ref(2)p has no effect in the female germ line, we expected no changes in density upon overexpression of either *cifA* or *cifB*. We overexpressed either *cifA* or *cifB* independently or together to determine their role in modulating *w*Mel density in stage-8 egg chambers. Overexpression of *cifB* resulted in a nonsignificant reduction of *w*Mel density of approximately 1.29-fold. Of note, this was similar to the level seen in autophagy knockdowns (see Fig. S6A, B, and E). Overexpression of either *cifA* alone or *cifA* and *cifB* together resulted in no change in *Wolbachia* density compared to that in the control. *cifA* alone displayed a fold change of 1, indicating no change in density (Fig. S6A, C, and E). *cifA* and *cifB* coexpression resulted in no significant increase in density (Fig. S6A, D, and E).

10.1128/mBio.02205-20.6 FIG S6 CifA and CifB overexpression do not affect *w*Mel density in the female germline. NGT;nos was used to overexpress Cif constructs. DNA is colored cyan, D cadherin (labeling the follicle cells) is yellow, and a fluorescently conjugated DNA probe detecting *Wolbachia* is in magenta. (A) Control stage-8 egg chambers show high germline *Wolbachia* densities. (B) Stage-8 egg chambers overexpressing CifB result in a nonsignificant trend for lower relative *w*Mel density. (C) Stage-8 egg chambers with overexpressed CifA result in a *Wolbachia* density similar to that in control egg chambers. (D) Stage-8 egg chambers overexpressing both CifA and CifB result in germline *Wolbachia* densities similar to that of the control. (E) A one-way ANOVA revealed a significant difference in the data set (*P* = 0.0171). A *post hoc* Tukey’s analysis revealed a significant difference between CifB overexpression and CifA-CifB overexpression groups (*N*^CifB^ = 30, *N*^CifA-CifB^ = 26, *P* = 0.024). Of note, CifB showed a trend for reduced *w*Mel density in the germline compared to that in the control (*N*^Cont^ = 27, *N*^CifB^ = 30, *P* = 0.0704). Grayscale panels display the *Wolbachia*-only channel. Scale bars, 40 µM. Download FIG S6, PDF file, 2.5 MB. Copyright © 2021 Deehan et al. 2021 Deehan et al. This content is distributed under the terms of the Creative Commons Attribution 4.0 International license.

### Global metabolomics identifies dysregulation of carbohydrate and glycerolipid metabolism in *Wolbachia*-infected autophagy mutant ovaries.

To attempt to address how autophagy may regulate *Wolbachia* density in the female germ line, we performed global metabolic profiling of autophagy knockdown and control ovaries in *w*Mel-infected and uninfected flies. Three replicates of four different samples, including uninfected wild type (w_Ctrl), uninfected Atg1 RNAi (w_mut), *w*Mel-infected wild type (mel_ctrl), and *w*Mel-infected Atg1 RNAi (mel_mut), were subject to liquid-liquid extraction tandem solid-phase microextraction (LLE-SPME) and run on Boston University’s Center for Network Systems Biology nanoscale liquid chromatography-mass spectrometry (nanoLC/MS) platform. Positive ion mode was used for sample comparison, and 10,251 features were detected in total displaying a metabolome drift (see Fig. S7A; Table S1). Principal-component analysis (PCA) revealed that PC1 accounts for 40% of the variation and clustered infected and uninfected ovaries well. PC2 accounted for 10% of the variation, showing a weak clustering of *w*Mel-infected ovaries with and without autophagy knocked down (Fig. S7B). The PCA showed that infection contributed mostly to the variance rather than the autophagy knockdown. Features were then implemented into MetaboAnalyst’s fast gene set enrichment analysis (fGSEA) platform to identify dysregulated pathways between samples (Table S1).

10.1128/mBio.02205-20.7 FIG S7 *Wolbachia* infection and autophagy knockdown drive different metabolic profiles. (A) Heat map indicating Z-score values for each detected positive ion mode metabolite. (B) Principal-component analysis of all positive ion mode-detected features. Download FIG S7, PDF file, 2.4 MB. Copyright © 2021 Deehan et al. 2021 Deehan et al. This content is distributed under the terms of the Creative Commons Attribution 4.0 International license.

10.1128/mBio.02205-20.8 TABLE S1 Positive ion metabolites and differentially regulated pathways. Download Table S1, XLSX file, 6.9 MB. Copyright © 2021 Deehan et al. 2021 Deehan et al. This content is distributed under the terms of the Creative Commons Attribution 4.0 International license.

Nine pathways were significantly dysregulated between *Wolbachia*-infected and uninfected ovaries, with seven pathways positively enriched and two pathways negatively enriched in *Wolbachia*-infected ovaries (Table S1). Pathways which can support central carbon metabolism were positively enriched and include pyruvate metabolism (normalized enrichment score [NES] = 1.946, *P* = 0.001), glycine, serine, and threonine metabolism (NES = 1.722, *P* = 0.005), butanoate metabolism (NES = 1.706, *P* = 0.006), propanoate metabolism (NES = 1.623, *P* = 0.017), citrate cycle (NES = 1.644, *P* = 0.019), d-glutamine and d-glutamate metabolism (NES = 1.562, *P* = 0.024), and glyoxylate and dicarboxylate metabolism (NES = 1.538, *P* = 0.026). Sphingolipid metabolism was negatively enriched (NES = −1.958, *P* = 0.003) and the pentose phosphate pathways was negatively enriched (NES = −1.510, *P* = 0.035). Interestingly, these data suggest central carbon metabolism is altered in *Wolbachia*-infected ovaries, with an increase in pyruvate metabolism and pathways which can support both pyruvate metabolism and the citrate cycle while reducing the pentose phosphate pathway, which compete with glycolysis for glucose-6-phosphate to generate nucleotides and NADPH (45).

**FIG 7:**
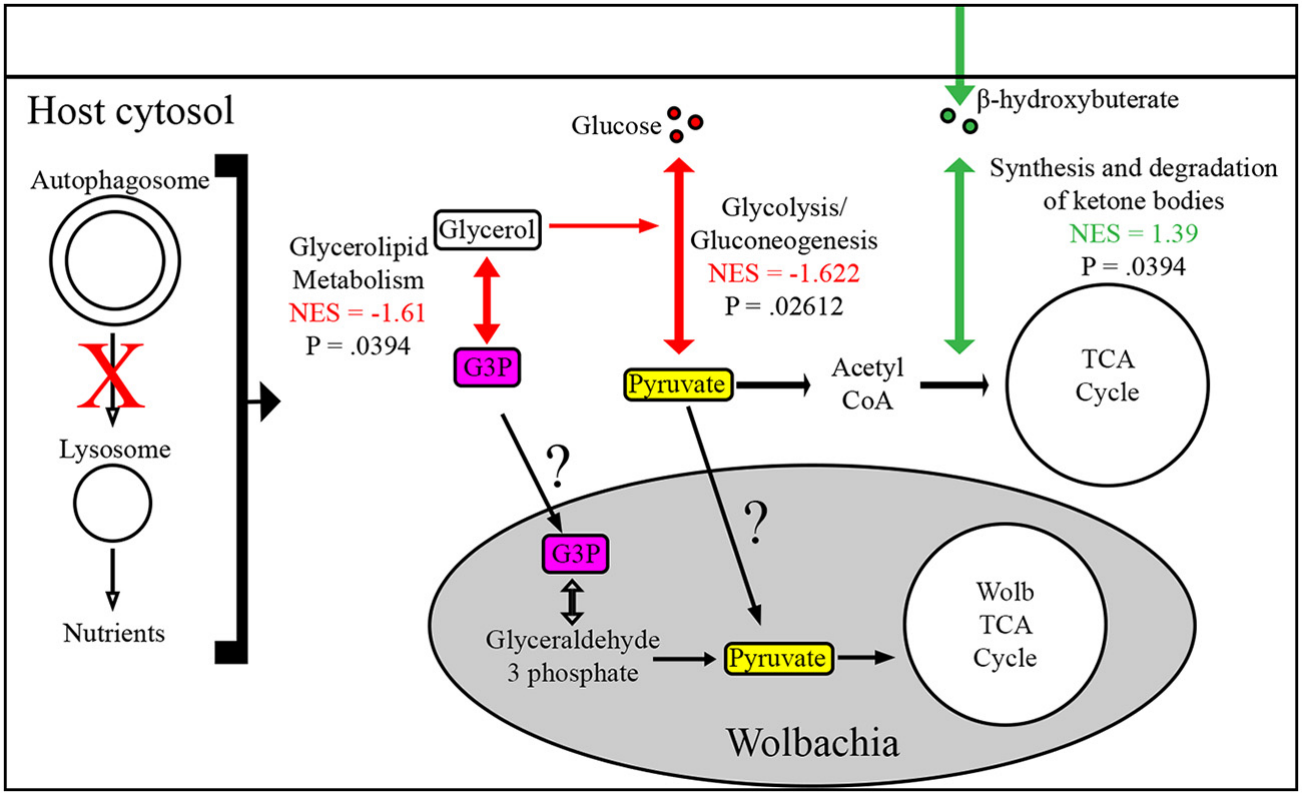
Differentially regulated metabolic pathways in Atg1 RNAi mutant ovaries of *w*Mel-infected flies in the context of a metabolic model. All significantly differentiated pathways are reported between *w*Mel-infected ovaries with autophagy knocked down (Atg1 RNAi) and the wild type. Downregulated metabolic pathways are highlighted with red, while upregulated pathways are green. Results are reported in the context of a proposed model of how they interact and could affect *Wolbachia*. NES, normalized enrichment score from fGSEA MetaboAnalyst results.

We investigated how knocking down autophagy in the presence of *Wolbachia* altered metabolic pathways which could lead to a decrease in *Wolbachia* density as previously seen. Under these circumstances, there were only three significantly dysregulated pathways, with one carbohydrate and two lipid metabolism pathways dysregulated (Table S1). Glycolysis (Fig. 7) (NES= −1.622, *P* = 0.026) was negatively enriched in autophagy mutant ovaries. For lipid metabolism, the synthesis and degradation of ketone bodies was positively enriched (Fig. 7) (NES = 1.39, *P* = 0.039) and glycerolipid metabolism was negatively enriched (Fig. 7) (NES = 1.61, *P* = 0.039). Interestingly, data suggest *Wolbachia* may compete for glycerol-3-phosphate and/or pyruvate from the host for energy, which are metabolites prevalent in our observed downregulated pathways (46, 47). Overall, these data reveal that autophagy mutants, when in the presence of *Wolbachia*, reduce glycolysis and glycerolipid metabolism, which could restrict *Wolbachia* density through limited accumulation of essential metabolites.

## DISCUSSION

Autophagy can act as an innate immune response aiding in the removal of pathogenic bacteria and viruses, but its role in host-endosymbiont interactions remains less understood. Previous results indicated that autophagy negatively regulated *Wolbachia* in systems predominantly composed of somatic cells, while known autophagy-inducing drugs increased *Wolbachia* density in the female germ line, leading to confusion in understanding the role of autophagy in modulating *Wolbachia* levels (16, 17). Here, we used systematic and comparative genetic approaches to address this discrepancy and determined that autophagy does modulate *Wolbachia* levels differentially in male somatic cells and female germ line cells. Moreover, we have identified bacterial proteins which modulate the interaction between *Wolbachia* and autophagy.

Our results show that Ref(2)p-mediated selective autophagy is responsible for negatively regulating *w*Mel but not *w*MelCS in the hub. Since *w*MelCS resides at naturally higher densities than *w*Mel, this result suggests that *w*MelCS evolved a mechanism to subvert host autophagy to aid in survival (discussed further below). These results do not completely agree with previous data that described *w*MelPop, a pathogenic strain more closely related to *w*MelCS than *w*Mel, being negatively related by autophagy. In that study, rapamycin treatment reduced *Wolbachia* density in whole larva, and Atg1 RNAi reduced density in *Drosophila* PC15 cells (16). For whole larval *in vivo* studies performed by Voronin et al. (16), the use of rapamycin, a TORC1 inhibitor, affects several different host-related pathways, including ribosome biogenesis, translation, and nutrient import, and has also been shown to stimulate ubiquitin-proteasome degradation (48). Proteasome degradation has been shown to support increased *Wolbachia* density, and this mechanism could possibly explain additional mechanisms by which rapamycin is capable of modulating several aspects of host biology which regulate *Wolbachia* accumulation (13). The close ties of autophagy and the ubiquitin proteasome should be teased apart in future studies to determine how each pathway exerts changes in *Wolbachia* density.

Beyond host biological mechanisms which could cause *Wolbachia* strain-specific density changes, bacterial derived differences could explain our observed strain-dependent subversion of host autophagy, including higher expression levels of bacterial effector proteins, which have been shown to aid in bacterial escape from autophagy (43). We tested a known functional effector of *w*Mel, CifB, which contains a ubiquitin-like protease domain (Ulp1) and has a moderate preference for lysine 63 ubiquitin chains (K63) over K48 (34, 49). Of note, K63 has been associated with P62-mediated selective autophagy in mammalian systems compared to K48, which is well characterized to be involved in proteolysis (50). Overexpression of CifA and CifB proteins individually showed CifB expression positively regulated *w*Mel density, while CifA expression showed a trend toward reducing *w*Mel density. When coexpressed, CifA and CifB showed no difference from the control. These results suggest that the deubiquitinating function of CifB could be protective for *Wolbachia* in cells in which autophagy negatively restricts higher densities, while CifA is antagonistic to CifB function, since CifA on its own showed a modest reduction in density and eliminated the benefit CifB expression had on *w*Mel density. These results partially agree with what has been reported previously (33). In the study by LePage et al. (33), qPCR of male testis overexpressing CifA or CifB in the germ line independently increased *Wolbachia* density, while overexpression of both constructs recapitulated CifA density increases. This observation disagrees with our CifA observation but agrees with our CifB observation and could be driven by a cell type-dependent interaction of host-bacterial proteins which can modify bacterial density that is still currently unknown.

Our follow-up CifB-autophagy epistasis analysis revealed that CifB functions in the autophagy pathway. When we expressed CifB or Atg1 RNAi individually, *w*Mel density increased. When coexpressed, density recapitulated Atg1 RNAi levels and not a summation of CifB and Atg1 RNAi levels. It should be noted that the hub can sustain extremely high densities of *Wolbachia* when infected with *w*MelPop (∼50× more than in surrounding tissue), even to the point of rupturing the hub cell plasma membrane, and so we believe modulating these pathways does not create a scenario where the hub cannot support an additive model density (37).

Biochemically, our data partially agree with what has been shown with the mosquito CifB homologue, CidB, for which extensive biochemical characterization has been done (34). Beckmann et al. (34) showed that when CidB is expressed in *Drosophila* males, it drives the cytoplasmic incompatibility (CI) phenotype, and this is abolished if CidB has a single amino acid change creating a catalytically dead DUB mutation. To biochemically characterize this protein’s function, *in vitro* assays and yeast studies were performed. CidB was shown to drive toxicity in yeast, and this was DUB domain dependent, as a catalytically dead mutant did not drive toxicity. CidA, the CifA homologue, was shown to bind CidB biochemically, and when expressed in yeast, it rescued CidB toxicity. This agrees with our model that CifB may rescue *w*Mel through its deubiquitinase activity and that CifA can block this. Interestingly, when *in vitro* ubiquitin cleavage assays were performed, CidB either alone or when coexpressed with CidA was capable of cleaving ubiquitin 48 (K48) or 63 chains, providing evidence that CidA does not directly block CidB DUB activity. Conversely, in yeast, the ubiquitin profile was not changed drastically when CidB was overexpressed, showing that further analysis must be completed to understand the exact function and that the result may be host or experiment specific (34, 35, 44, 49).

Autophagy has been implicated in male-derived sterility, with Ref(2)p homozygous mutants in *Drosophila* being described as male sterile (51). Our data implicating CifB in modulating Ref(2)p-mediated selective autophagy highlight a possible host-derived mechanism by which CI proteins drive sterility (33, 34, 36). From our studies, we are not proposing that CifA and CifB expression in the male hub directly affects male germ line Ref(2)p-mediated selective autophagy, but rather, our hub studies highlight CifB interacting with selective autophagy and that this should be explored in the male germ line. It should be recognized that the PD-(D/E)xK nuclease domains found in CifB, CidB, and other gene paralogs found in several *Wolbachia* strains are thought to be sufficient to drive CI in flies, indicating ubiquitinase activity may not be the only mechanism *Wolbachia* utilizes to drive CI (52).

Previous literature showed that *w*MelCS and *w*MelPop reside at higher densities than *w*Mel. *w*MelPop’s pathogenic overreplication has been elegantly shown to have a strong correlation with the octomom region of its genome, but it may still possess the capabilities to subvert autophagy *in vivo* similar to *w*MelCS (53). Since *w*MelCS is not regulated by Ref(2)p-dependent selective autophagy, this suggests that these closely related strains have evolved additional mechanisms to subvert the autophagy pathway. NCBI’s conserved domain database and previous genomic annotations of *Wolbachia* highlight *w*Mel as having several effector proteins which contain operational taxonomic unit (OTU) and Ulp1 domains, which have been shown to modulate host ubiquitination (31, 32, 41). WD0443, a protein which harbors an OTU domain, has a nonsynonymous amino acid change (R119C) between *w*Mel and *w*MelCS which could influence protein function (41). Additionally, an interesting candidate to study is the hypothetical *Wolbachia* protein WD0026, which has been predicted to be a secreted effector of *Wolbachia* and contains a Ulp1 domain (54). Expression level and/or coding differences in *Wolbachia* effector proteins may drive changes in host-*Wolbachia* interactions and should further be validated to begin to elucidate *Wolbachia*-host interactions and may be involved in host ubiquitin modulation and possibly autophagy subversion.

In the female germ line, a nutrient-sensitive tissue, autophagy positively regulated both *w*Mel and *w*MelCS. This was independent of Ref(2)p, implicating an association between bulk autophagy and *Wolbachia*. It should be noted that Ref(2)p RNAi achieved an 86% knockdown efficiency, and the remaining 14% expression could rescue an observed phenotype or indicate only modest reduction at the protein level; thus, this negative result should be interpreted with extra caution. Overall though, these results support previous data where flies fed rapamycin displayed higher density in the female germ line (17). Genetic analysis in that paper implicated a mechanism by which TOR signaling and possibly autophagy in surrounding follicle cells may modulate germ line *Wolbachia* density. Direct genetic modulation of autophagy was never performed in the female germ line or soma to determine if follicle cell autophagy modulated germ line *Wolbachia* density. Our results show direct evidence that cell-autonomous female germ line autophagy is capable of supporting strain-independent *Wolbachia* growth. Even though evidence pointed to bulk autophagy regulating *Wolbachia* in the germ line, we tested if CifA and CifB effectors could modulate density. Expression of CifA and CifB alone had no effect on *Wolbachia* density, but CifB expression did result in a trend for reduced *Wolbachia* density similar to autophagy knockdown. Coexpression of CifA and CifB also resulted in no difference in *Wolbachia* density. Since selective autophagy does not affect *Wolbachia* in the female germ line and CifB overexpression trends toward recapitulating the effect of autophagy knockdown, this may suggest CifB disrupts ubiquitin signaling involved in bulk autophagy (or a non-Ref(2)p-dependent form of selective autophagy) to target substrates for degradation and thus disrupts the beneficial effects autophagy provides for *Wolbachia.* Extensive biochemical analysis needs to be conducted to confirm which host ubiquitin substrate may be targeted by CifB.

Lastly, we performed global metabolomics to begin to identify what metabolic pathways may be dysregulated upon autophagy knockdown that are responsible for a reduction in *Wolbachia* density. It should be noted in the *Drosophila* female germ line autophagy is dispensable for proper egg formation, does not change fecundity, and does not influence egg hatching but remains active in the germ line, postulating that it may contribute to optimal metabolism in a metabolically demanding tissue (55). Previous metabolomics analysis of *Drosophila* ovaries revealed high levels of phosphoarginine, an energy reserve metabolite used to regulate ATP levels as well as increases in various lipid metabolites compared to that in other organs, highlighting increased lipid metabolism (56). In *Drosophila*, autophagy has been associated with glycogen breakdown in the fat body and amino acid metabolism through epigenetic modulation by a histone methyltransferase under starvation conditions in whole flies, but direct measurement of the metabolome in autophagy mutants has yet to be reported (57, 58). In RAS-driven cancer cell lines, glycolysis has been positively linked to autophagy, while in liver cancer cell lines, selective autophagy negatively regulates glycolysis through selective degradation of hexokinase 2 (57, 59, 60). These results highlight a complex relationship between these pathways which may be system and cell type dependent.

In *Wolbachia*-infected ovaries with autophagy knocked down, metabolomics analysis revealed a reduction in glycolysis/gluconeogenesis and glycerolipid metabolism. Interestingly, the density of *Wolbachia* infecting Brugia malayi has been coupled to host glycolysis and pyruvate levels, indicating that autophagy-induced reduction in glycolysis could lead to an unfavorable growth environment for *Wolbachia* (16, 47). It should also be noted that glycerol-3-phosphate is a member of the glycerolipid metabolism pathway, and *Wolbachia* has a predicted transporter and ability to convert glycerol-3-phosphate into a functional metabolite in *Wolbachia* glycolysis (46). Remarkably, there were no differentially regulated amino acid metabolism pathways, and *Wolbachia* has been shown to have extensive metabolism related to host amino acid sequestration. This does not rule out *Wolbachia* utilizing host amino acids as an energy source but, rather, supports additional host metabolites which *Wolbachia* may utilize.

**FIG 8:**
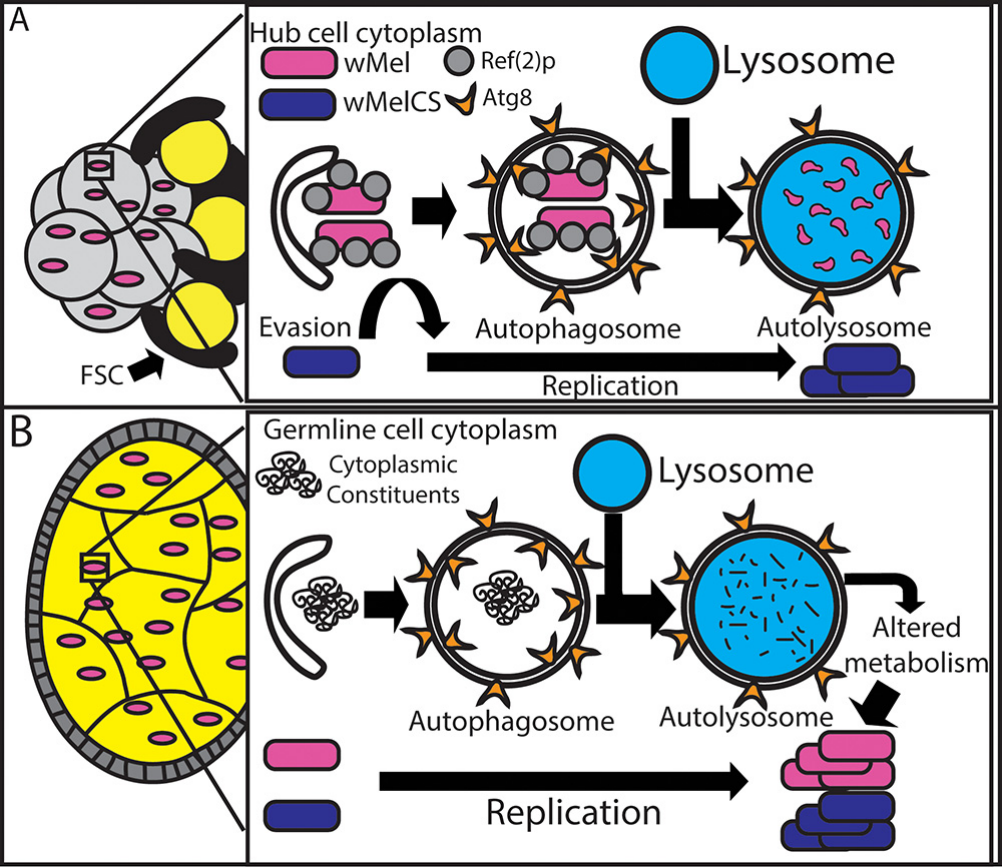
Model of how autophagy regulates *Wolbachia* density differently in the germ line and somatic cell types. (A) Depiction of how autophagy regulates *Wolbachia* density in the hub in a strain-dependent manner. *w*Mel is shown to be negatively regulated by selective autophagy. (B) In the germ line, *Wolbachia* is able to subvert Ref(2)p-mediated selective autophagy. Knockdown of autophagy proteins involved in bulk autophagy results in a decrease in *Wolbachia* density. This indicates a possible mechanism by which *Wolbachia* utilizes autophagy-derived nutrients for energy.

The data described here support a working model for fundamental understandings that cell type can define the role autophagy has in interacting with intracellular microbes (Fig. 8). In static cell types where selective autophagy is the predominant autophagy pathway, bacteria may be negatively impacted if they are recognized by the host and subsequently targeted for degradation. In dynamically growing cell types which are highly nutrient sensitive, such as the developing eggs of *Drosophila* females, the bulk autophagy pathway may be predominant. These underlying biological characteristics of cell types have rarely been addressed both in the host-pathogen and host-endosymbiont fields. We provide additional evidence for the role of deubiquitinating enzymes in bacterial survival within a host cell. Specific to the *Wolbachia* field, CifA and CifB are essential proteins to study because of their ability to drive CI, a highly parasitic reproductive phenotype which is imperative for establishing *Wolbachia*-infected mosquitoes in the wild. CI provides a selective advantage for the establishment of *Wolbachia*-infected mosquitoes which are then capable of reducing human pathogens such as Zika and dengue viruses. Our characterization of CifA and CifB and identification of CifB functions in the autophagy pathway are important to further understand how these proteins could interact with Ref(2)p in driving male sterility and to modulate intracellular bacterial titers, which may indirectly play a role in pathogen blocking through boosting *Wolbachia* density rather than interacting directly with the pathogens (3–5, 51).

## MATERIALS AND METHODS

### Fly maintenance and stocks.

For information on specific fly strains, including their full genotype and source reference, see Table S2 in the supplemental material. All fly crosses were maintained at 25°C and reared on a mixture of molasses, cornmeal yeast, and agar supplemented with active dry yeast pellets. For knockdown of autophagy in the hub and polar cells, *Wolbachia*-infected virgin females with the unpaired (Upd) Gal4 driver (Upd;;) were crossed to males with an autophagy RNAi construct. Autophagy RNAi constructs were balanced with either curly (CyO), tubby humeral (TM6B), or MKRS as indicated in Table S2. In the germ line, *Wolbachia*-infected females had the nanos Gal4-tubulin and nanos Gal4 drivers on the second and third chromosomes, respectively (;NGT;nos). Virgin females were crossed to males which had UAS-autophagy RNAi constructs balanced as previously described. In both soma and germ line crosses, F1 flies inheriting the balancer were used as a control to which the autophagy knockdown siblings were compared, allowing for flies from the same parental cross to be compared. F1 offspring were allowed to age 7 days before testis or ovary dissection. In germ line analysis, 10 control and 10 experimental female flies were housed in vials with 10 male flies, and flies were flipped to new food on day 4. Subsequently, tissue was fixed, stained, and imaged according to descriptions below.

10.1128/mBio.02205-20.9 TABLE S2 Drosophila fly stock genotypes used in experiments. Download Table S2, XLSX file, 0.01 MB. Copyright © 2021 Deehan et al. 2021 Deehan et al. This content is distributed under the terms of the Creative Commons Attribution 4.0 International license.

### Dissection, fixation, and staining protocols for cell types and detecting *Wolbachia*.

Tissues were dissected and fixed for 20 min in a 4% formaldehyde solution (EMS) with Grace’s insect medium (Lonza catalog no. 04-457F) and 0.2% Triton X-100 (Sigma-Aldrich). For the autophagy knockdown hub analysis (Fig. 1), antibody staining was performed according to references 37 and 61. Mouse anti-HSP60 antibody (1:100; Sigma-Aldrich) was used to detect *Wolbachia*, and rat anti-D cadherin antibody (DCAD2, concentrated, 1:200; DSHB) was used to visualize the hub. Alexa Fluor secondary antibodies were used to visualize primary antibodies (Invitrogen). Cif hub experiments and female germ line experiments utilized a modified antibody *in situ* protocol previously described in reference 62. The rat anti-D cadherin antibody was used to mark the hub and, in the female germ line, the boundary between somatic cells and germ line cells. In germ line staining, control and experimental tissues were dissected and fixed and underwent antibody staining in separate tubes. Different secondary antibody fluorophores were used (goat anti-rat IgG 488 [experimental], goat anti-rat IgG 633 [control]) to later identify experimental and control tissues. After antibody staining, control and experimental tissue were combined to be subject to the same exact *in situ* protocol for *Wolbachia* detection. Two *Wolbachia* probes labeled with Cy3 at the 5′ end were used: Wpan16S887 5′-ATCTTGCGACCGTAGTCC-3′ and Wpan16S450 5′-CTTCTGTGAGTACCGTCATTATC-3′. Hybridization was performed at 37°C in 50% formamide, 5× SSC (1× SSC is 0.15 M NaCl plus 0.015 M sodium citrate), 250 mg/liter salmon sperm DNA, 0.5× Denhardt’s solution, 20 mM Tris-HCl, and 0.1% SDS. After a 30-min preincubation period, tissue was incubated in 100 ng of each probe for 3 h. Tissue was then washed twice for 15 min at 37°C in a 1× SSC wash with 0.1% SDS and 20 mM Tris-HCl and then twice for 15 min in a 0.5× SSC wash with 0.1% SDS and 20 mM Tris-HCl. Hoechst stain was added to all the posthybridization washes at a concentration of 10 µg/ml. Tissue was then washed in phosphate-buffered saline (PBS), mounted in Prolong Gold antifade solution, and imaged as described below.

### Image acquisition and quantification of autophagy’s effect on *Wolbachia*.

A FluoView FV1000 confocal microscope system (Olympus) was utilized to acquire images for subsequent analysis. Laser power, sensitivity (HV), gain, offset, and Kalman filter paramters were the same for control and experimental images of the same data set. One-micron z-stack images were acquired of entire hubs or egg chambers. Images were taken at ×600 magnification (60× lens objective) For hubs; a 2.6 digital zoom was implemented for better visualization. Relative *Wolbachia* density was quantified by imaging the cell type of interest (COI; hub or female germ line) and surrounding cells (SC). The SC in the hub consist of the mitotic region of the germ line. The SC of the germ line were the surrounding follicle cell layer. For hubs, all middle z-stack slices were quantified and normalized to the surrounding cell type density. This was to ensure correct quantification of all *Wolbachia* organisms within our COI, since *Wolbachia* infection and density can vary cell to cell and from individual to individual. For the germ line, stages 2 to 8 were identified, and the middle 5 z-stack planes were used for analysis (63). The calculation was as follows:

$$\frac{\text{COI voxel density}(\text{pixel intensity of }\mathrm{Wolbachia}/\text{pixel area of COI})}{\text{SC voxel density}(\text{pixel intensity of }\mathrm{Wolbachia}/\text{pixel area of SC})}.$$

### Nucleic acid purification.

For all nucleic acid purifications, ovaries were dissected and placed in an empty 1.5-ml Eppendorf tube and stored at −80°C until extraction. DNA was purified utilizing the Qiagen blood and tissue kit (catalog number [no.] 69506) per the manufacturer’s protocol. In brief, dissected and homogenized tissues were treated with proteinase K for 3 h, column purified, and eluted in 100 μl of molecular-grade water. RNA was purified by either the Qiagen miRNeasy minikit (catalog no. 217004) to test Atg1 RNAi knockdown or TRIzol for all subsequent experiments. For TRIzol extractions, 150 μl of TRIzol was added to a 1.5-ml Eppendorf tube with samples and homogenized. Pestles were washed with 850 μl of TRIzol to recover any sample tissue attached to the pestle. Samples were spun at 4°C for 15 s at 12,000 × *g*. Afterwards, 200 μl of 100% chloroform was added and briefly vortexed. Samples were centrifuged at 4°C for 15 min at 20,000 × *g*. The transparent supernatant was removed and transferred to a new 1.5-ml Eppendorf tube. Isopropanol (100%) was added 1:1 (vol/vol) and mixed by inversion. Subsequently, 1 μl of 20 μg/μl GlycoBlue was added to each sample and incubated for 30 min at −80°C. Samples were immediately centrifuged at 4°C for 15 min at 20,000 × *g*. The supernatant was removed, and 200 μl of 75% ethanol was used to resuspend the pellet. Samples were centrifuged at 4°C for 5 min at 7,500 × *g*. The supernatant was removed, and pellets were able to air dry for no longer than 10 min. Samples were resuspended in 50 μl of RNase/DNase-free sterile molecular-grade water and incubated for 10 min at 55°C. RNA was then treated with a Turbo DNA-free kit (AM1907) according to the manufacturer’s protocol. Samples were stored at −80°C.

### Quantitative PCR and quantitative reverse transcriptase PCR.

qPCR was used to detect *Wolbachia* density and RT-qPCR was used to determine knockdown efficiency of RNAi constructs in ovaries. Pools of 5 female ovary pairs were combined per experimental replicate for all quantitative experiments. An EXPRESS one-Step SYBR GreenER kit with premixed ROX from Life Technologies, Inc., was used (catalog no. 1179001k) to detect DNA (reverses transcriptase removed) and mRNA (reverse transcriptase included). Ten nanograms of either DNA or mRNA was added to reaction mixtures. Primers for *Wolbachia* detection were wsp_F, 5′-TTGGAACCCGCTGTGAATGA-3′, and WSP_R, 5′-CCGAAATAACGAGCTCCAGCA-3′, which were normalized to the host gene 14-3-3 using primers 14-3-3F, 5′-CATGAACGATCTGCCACCAAC-3′, and 14-3-3R, 5′-CTCTTCGCTCAGTGTATCCAAC-3′. For autophagy knockdown confirmation, autophagy-specific primers were picked from reported primers designed by the Drosophila RNAi Screening Center (DRSC). The following primers were used: Atg1_F, 5′-CGTCAGCCTGGTCATGGAGTA-3′; Atg1_R, 5′-TAACGGTATCCTCGCTGAG-3′ (DRSC, PA60369); ATG8a_03F, 5′-GGTCAGTTCTACTTCCTCATTCG-3′; ATG8a_03R, 5′-GATGTTCCTGGTACAGGGAGC-3′; Ref(2)p_F, 5′-AATCGAGCTGTATCTTTTCCAGG-3′; and Ref(2)p_R, 5′-AACGTGCATATTGCTCTCGCA-3′. For internal normalization, primers Rpl32_F, 5′-ATGCTAAGCTGTCGCACAAATG-3′, and Rpl32_R, 5′-GTTCGATCCGTAACCGATGT-3′, were used (64). Technical replicate average threshold cycle (*C_T_*) values for all qPCR experiments can be referenced in Table S3.

10.1128/mBio.02205-20.10 TABLE S3 Average *C_T_* values in qPCR experiments. Download Table S3, XLSX file, 0.01 MB. Copyright © 2021 Deehan et al. 2021 Deehan et al. This content is distributed under the terms of the Creative Commons Attribution 4.0 International license.

### Metabolomic sample preparation, LC-MS analysis, and data processing.

Forty total ovaries (20 female flies under each condition) were extracted by cold methanol-acetonitrile-water (MeOH-ACN-H_2_O; 40/40/20 [vol/vol]), cleaned up/enriched by SPME, and dried using a vacuum concentrator. Afterwards, metabolites were reconstituted in 2% ACN before injection. A C_18_ precolumn (3 μm, 100 Å, 75 μm by 2 cm) hyphenated to a rapid separation liquid chromatography (RSLC) C_18_ analytical column (2 μm, 100 Å, 75 μm by 25 cm) was used to separate metabolites. LC-MS/MS analyses were completed using an EASY nLC 1200 system (Thermo Scientific) coupled to a Q Exactive HF mass spectrometer. Full MS spectra were collected at a unit resolution of 60,000 with an automatic gain control (AGC) of 3 × 10^6^ or maximum injection time of 25 ms and a scan range of 67 to 1,000 *m/z*. MS2 scans were performed at a resolution of 15,000 and using stepped normalized collision energy of 10, 20, and 40 (%). Source ionization parameters were optimized with the spray voltage at 1.8 kV, dynamic exclusion was set to 10 s. Raw data were converted to mzML files with msConvert, and peak detection, deconvolution, and retention time alignment were performed using OpenMS. Subsequently, the *m/z* to pathway was enriched by MetaboAnalyst 4.0.

### Statistics and graphing.

All statistics were performed in R, and a *P* value of <0.05 was considered significant. For hub analysis, data were checked for normality by performing the Shapiro-Wilks test and determined to not be normally distributed. For pairwise comparisons of two unrelated samples, the Mann-Whitney U test was performed. For multigroup comparisons, a Kruskal-Willis test was performed, and if found significant, a *post hoc* Dunn’s test with Bonferroni corrections was performed to determine which groups were significantly different. For the germ lines, all data were checked for normality with the Shapiro-Wilks test and found to be normally distributed. Unpaired Student’s *t* tests were performed to compare two independent samples. In the case of multigroup comparisons, a one-way analysis of variance (ANOVA) was performed, and when significant, a *post hoc* Tukey’s test was performed to identify statistically significant groups. All graphs were made in R using the raincloud theme.
