# Supplementary figures and images for: Intracellular Density of Wolbachia Is Mediated by Host Autophagy and the Bacterial Cytoplasmic Incompatibility Gene cifB in a Cell Type-Dependent Manner in Drosophila melanogaster
Source: mBio. 2021 Jan 12;12(1):e02205-20. doi: 10.1128/mBio.02205-20 (PMC7844536; doi:10.1128/mBio.02205-20)

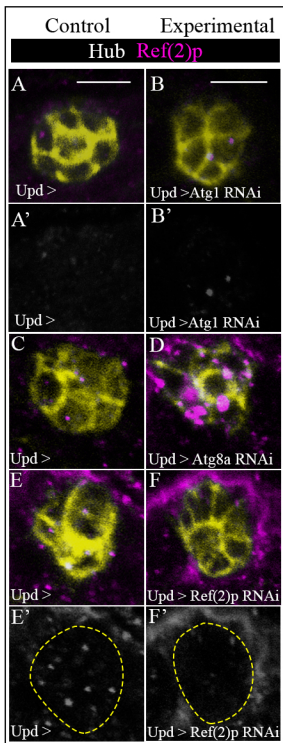

Supplement: FIG S1 [file mBio.02205-20-sf001.pdf]

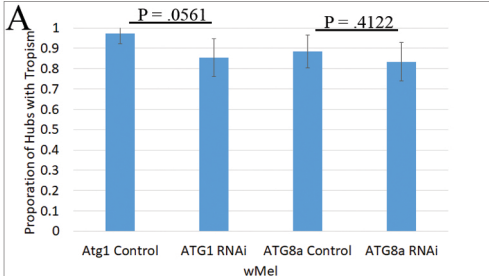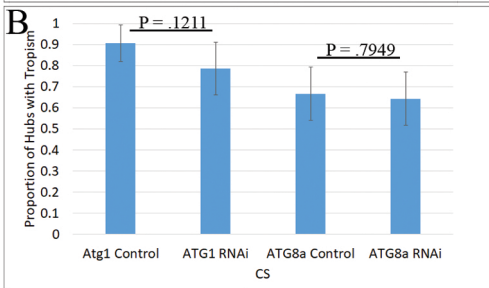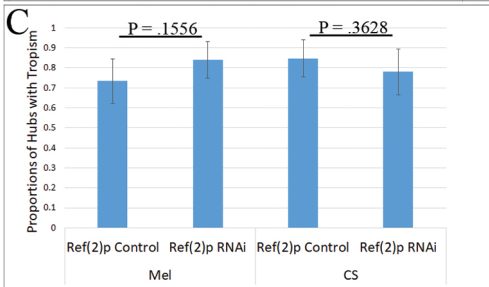

Supplement: FIG S2 [file mBio.02205-20-sf002.pdf]

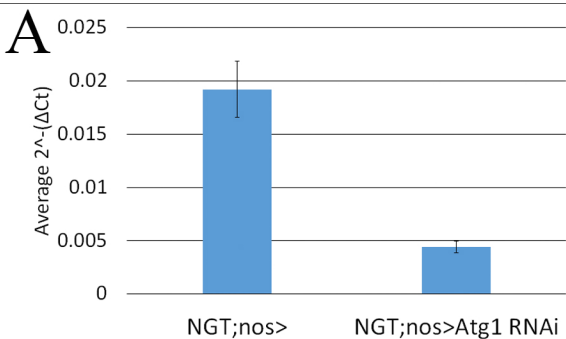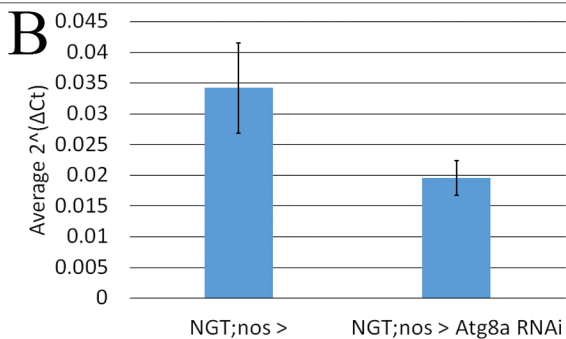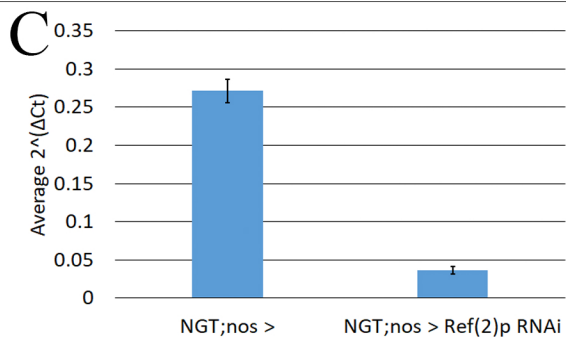

Supplement: FIG S3 [file mBio.02205-20-sf003.pdf]

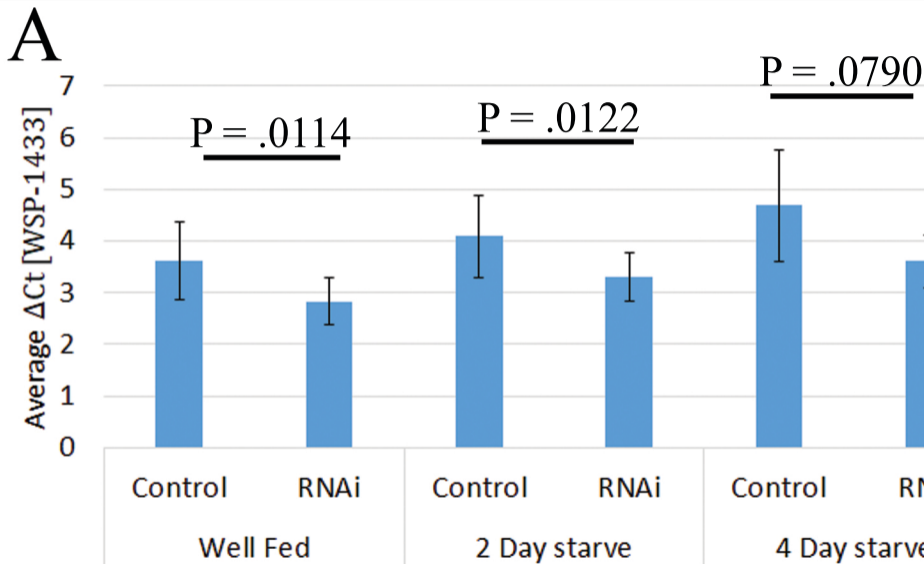

Supplement: FIG S4 [file mBio.02205-20-sf004.pdf]

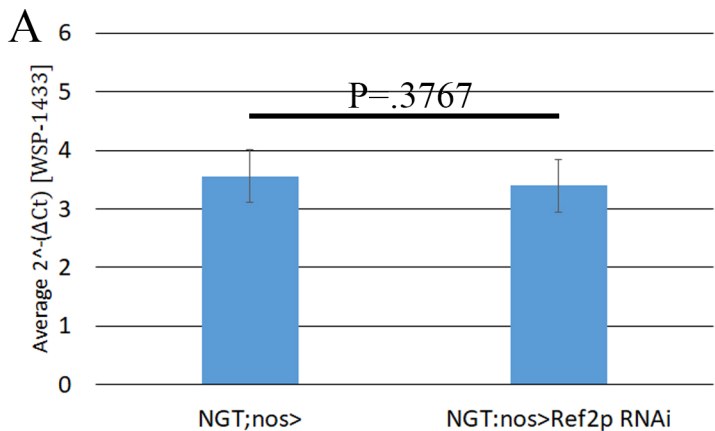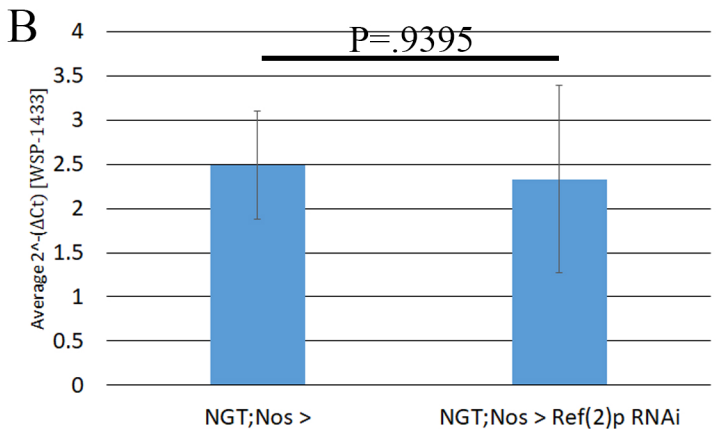

Supplement: FIG S5 [file mBio.02205-20-sf005.pdf]

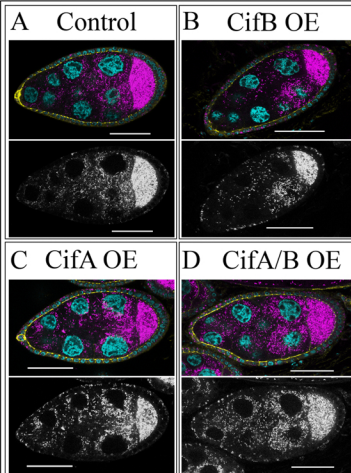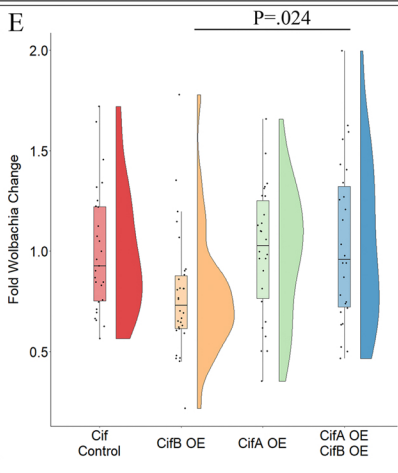

Supplement: FIG S6 [file mBio.02205-20-sf006.pdf]

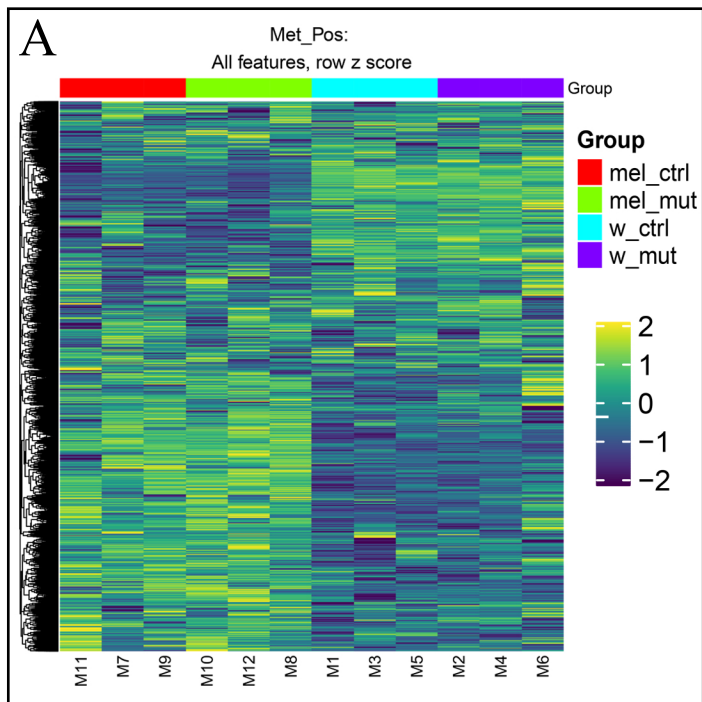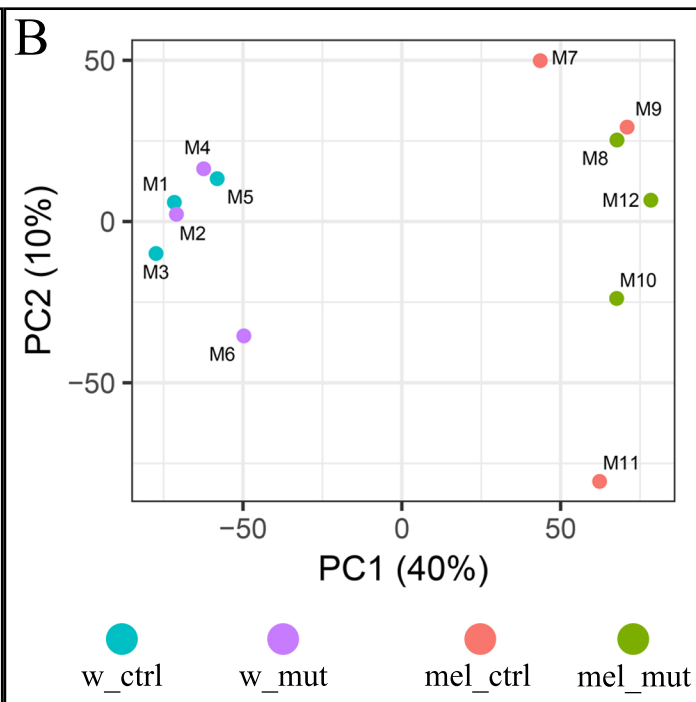

Supplement: FIG S7 [file mBio.02205-20-sf007.pdf]
